# The microtubule cytoskeleton: An old validated target for novel therapeutic drugs

**DOI:** 10.3389/fphar.2022.969183

**Published:** 2022-09-15

**Authors:** Laurence Lafanechère

**Affiliations:** Team Cytoskeleton Dynamics and Nuclear Functions, Institute for Advanced Biosciences, Université Grenoble Alpes, INSERM U1209, CNRS UMR 5309, Grenoble, France

**Keywords:** microtubules, tubulin-binding drugs, targeted therapies, microtubule regulations, tubulin post-translational modifications, drug development, tubulin

## Abstract

Compounds targeting microtubules are widely used in cancer therapy with a proven efficacy. However, because they also target non-cancerous cells, their administration leads to numerous adverse effects. With the advancement of knowledge on the structure of tubulin, the regulation of microtubule dynamics and their deregulation in pathological processes, new therapeutic strategies are emerging, both for the treatment of cancer and for other diseases, such as neuronal or even heart diseases and parasite infections. In addition, a better understanding of the mechanism of action of well-known drugs such as colchicine or certain kinase inhibitors contributes to the development of these new therapeutic approaches. Nowadays, chemists and biologists are working jointly to select drugs which target the microtubule cytoskeleton and have improved properties. On the basis of a few examples this review attempts to depict the panorama of these recent advances.

## Introduction

Microtubules (MTs) were first observed under a polarizing microscope in the mid-1960s as the birefringent fibrils, which constitute the mitotic spindle responsible for the correct segregation of chromosomes into the two daughter cells [for an historical review see (Inoué, 1981)]. In addition to this central role in cell division, MTs are also crucially involved in other cellular functions such as the establishment and maintenance of cell morphology and motility. They are central players of intracellular trafficking and serve as rails for molecular motors of the dynein and kinesin families that transport protein complexes or vesicles over long distances (Barlan and Gelfand, 2017; Lee Sweeney and Holzbaur, 2018). These movements are responsible for the characteristic distribution of the endoplasmic reticulum, the Golgi apparatus and other organelles in the cytoplasm. More recently, a role of MTs in neuronal polarity and synaptic plasticity has been revealed (Hoogenraad and Bradke, 2009; Jaworski et al., 2009). Because of their involvement in these key cellular activities, compounds that interfere with the function of the microtubule cytoskeleton have been developed as cytotoxic agents to combat parasites and cancer. Moreover, the discovery of their contribution to synaptic plasticity opens the door to new therapeutic opportunities in the field of neuronal diseases (Andrieux et al., 2006; Baas and Ahmad, 2013; Eira et al., 2016; Brunden et al., 2017; Sferra et al., 2020; Jamenis et al., 2022).

Given the multiple cellular functions of MTs and their presence in all cell types, their therapeutic targeting consequently induces, unfortunately, numerous undesirable side effects such as peripheral neuropathies, neutropenia, gut toxicity, alopecia. Moreover, the potency of clinically used microtubule inhibitors is limited by the emergence of resistances (Dumontet and Jordan, 2010; Yang and Horwitz, 2017).

With regard to cancer treatment, despite therapeutic innovations such as immunotherapies or targeted therapies (i.e., therapies that target the mechanisms by which cancer cells develop or spread)**,** the importance of microtubule-targeting drugs (MTD) remains however undiminished. Given that MTs are essential for the process of mitosis, the long-held idea was that clinically used MTD act mainly by decreasing the rate of excess proliferation which has been described as an important feature of cancer progression (Komlodi-Pasztor et al., 2011). With the hope to lower the neurotoxicity, which is a common undesirable side-effect of these drugs, considerable efforts have been made to develop drugs targeting proteins other than tubulin, that are essential for mitosis. Thus, potent and selective inhibitors of mitotic kinases, such as AurkA, AurkB, and Plk1, or mitotic motors such as kinesin-5, CenpE, have been developed. Unfortunately, these drugs proved to be a clinical failure, as they induced severe neutropenia or had intestinal toxicity at doses well below the therapeutic dose (Komlodi-Pasztor et al., 2012; Marzo and Naval, 2013).

The clinical failure of mitosis-specific drugs contrasts sharply with the continuing clinical success of drugs that target microtubules, which include the vinca alkaloids, taxanes, ixabepilone (a semi-synthetic analog of epothilone B) and eribulin (Dumontet and Jordan, 2010; Steinmetz and Prota, 2018).

Many research efforts are being undertaken to understand the therapeutic mechanism of action of these MTD. In particular, proliferation measurements of cancer cells in tumors indicate that they divide much more slowly than previously thought. The selective targeting of cells in mitosis is therefore not compatible with a therapeutic action: the proliferation rate paradox has been mentioned (Mitchison, 2012). Do MTD act by targeting dividing cancer cells and the dying cells in turn induce an environmental change leading to the destruction of neighboring cancer cells, a so-called bystander effect, which would allow to target more tumor cells (Mitchison, 2012)? Do these drugs perturb other MT functions, especially in interphase (Wang et al., 1998; Giannakakou et al., 2000; Stone and Chambers, 2000; Ogden et al., 2014; Mitchison et al., 2017; Smith et al., 2021)?

Other important research efforts focus on the mechanisms leading to the adverse effects of MTD, particularly peripheral neuropathies. In fact, not only agents targeting MTs, but also antitumor agents with very different mechanisms of action, such as DNA cross-linking, proteasome inhibition and immunomodulation, induce peripheral neuropathies. Is this the result of a shared convergent or of a different mechanism of action?

The detailed characterization of the mechanisms leading to tumor resistance against MTD is another current field of research. Clearly, the more finely we dissect the role of microtubules and the cytoskeleton in cellular functions, the more we will be able to target diseases differentially and avoid side effects.

With the emerging knowledge, chemists and biologists are now working jointly to select drugs which target the MT cytoskeleton and have improved properties. The aim of this review is not to give an exhaustive summary of all current developments, but rather to paint a picture of recent advances on the basis of a few examples (Table 1).

**TABLE 1:** Chemical compounds cited in this article, listed (alphabetically) according to their mode of action.Regarding therapeutic indications, a distinction has been made between drugs approved or in clinical trials (no question mark, source PubChem, https://pubchem.ncbi.nlm.nih.gov/) and drugs with potential applications (question marks) according to experimental data on cells or animal models.

Compounds	Target(s)	Effect on microtubules	Specific features	Therapeutic indication(s)	Main references cited in this review
**Tubulin is the target**					
**Benzimidazoles**	Tubulin (colchicine site)	Destabilization	Many of these compounds show selectivity for βVI tubulin isotype	Hematologic cancers?	Montecinos et al. (2022)
**Carba1**	Tubulin (colchicine site)	Destabilization	Synergizes with paclitaxel	Cancer?	Peronne et al. (2020)
**Colchicine**	Tubulin (colchicine site, at the intradimer interface)	Destabilization	Accumulates in hepatocytes, where MT are depolymerised	Inflammatory diseases	Steinmetz and Prota, (2018); Giraudel et al. (1998); Ravelli et al. (2004); Dorléans et al. (2009); Weng et al. (2021)
**Dolastatin**	Tubulin (vinca site on β-tubulin)	Destabilization	Used in cancer treatment, as antibody-drug conjugate	Cancer	Chen et al. (2017)
**Eribulin**	Tubulin (vinca site on β -tubulin)	Destabilization		Cancer	Dumontet and Jordan, (2010); Steinmetz and Prota, (2018)
**Gatastatin**	γ-tubulin	Inhibition of nucleation	Only cytotoxic when in combination with PLK1 inhibitors	Cancer?	Chinen et al., 2015 Shintani et al., 2020; Ebisu et al. (2021)
**Gatorbulin-1**	Tubulin (gatorbulin site, at the intradimer interface)	Destabilization	First ligand of a new site on tubulin	Cancer?	Matthew et al. (2021)
**Ixapibelone**	Tubulin (taxanes site on β-tubulin)	Stabilization	Active on cells overexpressing b-III tubulin	Cancer	Dumontet et al. (2009)
**Laulimalide/Peloruside**	Tubulin (laulimalide/peloruside site on β-tubulin	Stabilization		Cancer?	Steinmetz and Prota, (2018)
**Maytansine**	Tubulin (maytansine site on β-tubulin)	Destabilization	Used in cancer treatment, as antibody-drug conjugate	Cancer	Steinmetz and Prota, (2018); Nicoletti et al. (2015)
**Paclitaxel**	Tubulin (taxanes site on β-tubulin)	Stabilization		Cancer	Dumontet and Jordan, (2010); Steinmetz and Prota, (2018); Perone et al. (2021)
**Pironetin**	Tubulin (pironetin site on α-tubulin)	Destabilization	Only known pure α-tubulin ligand. The binding is covalent	Cancer?	Usuis et al. (2004); Yang et al. (2016b); Steinmetz and Prota, (2018)
**Todalam**	Tubulin (todalam site, located partly on α- and β-tubulins, between two longitudinally aligned dimers	Destabilization	First ligand of a new site on tubulin	Cancer?	Mühlethaler et al. (2022)
**Vinca alkaloids**	Tubulin (vinca site on β-tubulin)	Destabilization		Cancer	Dumontet and Jordan, (2010); Steinmetz and Prota, (2018)
**Selective targeting of parasite tubulin**					
**Parabulin**	Tubulin of Apicomplexa	Destabilization	Toxic for apicomplexan parasite *T. gondii,* without harming the human host cells	Malaria? Toxoplasmosis?	Gaillard et al. (2021)
**Kinase inhibitors with tubulin as an independent off-target**					
**Buparlisib**	PI3K and tubulin (at the colchicine site)	Destabilization		Cancer	Bohnacker et al. (2017)
**LIMKi 2, LIMKi5**	LIM Kinase and tubulin	Destabilization			Ross-Macdonald et al. (2008)
**Masitinib**	TKR and tubulin (indirectly)	Stabilization		Amyotrophic lateral sclerosis, Mastocytosis	Ramirez-Rios et al. (2020)
				Pancreatic cancer Gastrointestinal stromal tumor	
				Dog mast-cell tumors	
**Nintedanib**	TKR and tubulin (indirectly)	Stabilization		Idiopathic pulmonary fibrosis Recurrent non-small cell lung cancer (with docetaxel)	Ramirez-Rios et al. (2020)
**Nocodazole**	ABL, c-KIT, BRAF, MEK and tubulin (at the colchicine site)	Destabilization	Mainly used as a tool to study microtubule functions		Park et al. (2012)
**Rigosertib**	PLK1 and tubulin (at the colchicine site)	Destabilization		Cancer	Jost et al. (2017)
**Selonsertib**	ASK1 and tubulin (indirectly)	Stabilization		Non-alcoholic steatohepatitis	Ramirez-Rios et al. (2020)
**Tivantinib**	MET receptor tyrosine kinase and tubulin (at the colchicine site)	Destabilization		Hepatoblastoma	Basilico et al. (2013); Katayama et al. (2013); Aoyama et al. (2014); Wang et al. (2016)
**Other drugs with tubulin as an independent off-target**					
**Bortezomib**	26S proteasomal subunit and tubulin	stabilization	26S proteasomal subunit and tubulin are independent targets	Multiple myeloma Mantle cell lymphoma	Staff et al. (2013); Meregalli et al. (2014); Malacrida et al. (2021); Pero et al. (2021)
**Clozapine**	Monoamine receptors and tubulin	destabilization	Monoamine receptors and tubulin are independent targets	Schizophrenia	Hino et al. (2022)
**Drug that targets MT regulators**					
**Pyr1**	LIM Kinase (LIMK)	stabilization	MTs stabilization results from LIMK inhibition	Cancer? Schizophrenia?	Prudent et al. (2012); Prunier et al. (2016a); Gory-Fauré et al. (2021)

## Characterization of tubulin drug binding sites: Targeting microtubules, but differently

### At first, there were six different drug binding sites

MT-targeting agents can be broadly divided into microtubule-stabilizing, for instance taxanes and epothilones, and microtubule-destabilizing agents, for instance vinca alkaloids, colchicine and eribulin. Six different binding sites on the αβ-tubulin heterodimer have been described: four sites (Vinca, Taxane, Maytansine and Laulimalide/Pelorusite sites) on the β-subunit, one site (Colchicine site) at the tubulin intradimer interface (Giraudel et al., 1998; Ravelli et al., 2004; Dorléans et al., 2009) and one site (Pironetin site) on the α-subunit (Usui et al., 2004; Yang et al., 2016b). Although these compounds all target tubulin, they have different therapeutic applications, reinforcing the interest in finding new tubulin-targeting drugs.

Here, I present recent approaches that have identified new binding sites on tubulin, and methods to identify drugs that target these binding sites.

### And then, there were seven

The first approach (Matthew et al., 2021), carried out by Hendrik Luesch’s team in the Department of Medicinal Chemistry at the University of Florida in the United States, looked for new drugs by exploring the variety of natural substances. They collected several samples of the prolific marine cyanobacterium *Lyngbya* cf. *confervoides* off the coast of Florida. They then made extracts from these cyanobacteria, which they fractionated by HPLC and the fractions were tested for antiproliferative properties on colon cancer cells. This allowed to isolate a cytotoxic compound, of which they determined the structure to be a cyclodepsipeptide, that they named Gatorbulin-1. The concentration of Gatorbulin-1 that inhibits cell viability by 50% (IC_50_) was found to be in the sub-micromolar rang (0.80 μM). Then, to prove the structure and overcome the supply issue, which is a common problem for natural substances, they embarked on total synthesis of the compound and succeeded to obtain Gatorbulin-1 from epoxysuccinic acid in 20 steps, with an overall yield of 5.6%. After overcoming these initial obstacles of identifying and fully synthesizing the active compound, they took on the major challenge of identifying the compound’s target and mechanism of action, through a rigorous “funnel” strategy, starting from the analysis of the effect of the compound on a large number of cell lines, indicating a cytotoxicity profile mainly related to antimitotic agents/tubulin, then with the analysis of its effect on cellular microtubules by immunofluorescence and finally using pure tubulin for *in vitro* assembly assays. They demonstrated that Gatorbulin-1 directly inhibits tubulin polymerization *in vitro*. Then, in collaboration with the Madrid team of F. Diaz, they obtained the structure of tubulin in association with the compound, which revealed a new drug binding site on tubulin, at the intradimer interface of tubulin, as it is the case for colchicine. Thus, this integrated approach allowed not only to enrich basic knowledge on the structure of tubulin, with the discovery of a new, unexplored, binding site, but also the discovery of a promising small-molecule, with a distinct chemotype, drug-like properties and translational potential.

### And now there could be twenty or more

The second approach was conducted in M. Steinmetz’s team, at the Paul Scherrer Institute in Switzerland. Using molecular dynamics simulation with a high-resolution crystal structure of the αβ-tubulin heterodimer, coupled to a computational analysis, this team identified several pockets (i.e., predicted binding sites) on the β-subunit and on the α-subunit. Interestingly, by tracking the exchange of atoms between adjacent pockets during the course of the simulation, they identified communication networks between these pockets, that predict crosstalk between different sites.

Since this team has developed a robust and well-established αβ-tubulin heterodimer crystallization system (Prota et al., 2013), they then conducted an X-ray crystallography-based screen, by soaking individual crystals with a library of several hundred of different small chemical entities, called fragments. After X-ray diffraction, they solved the structure of most of these complexes and were able to identify 56 chemically diverse fragments that target a total of 10 different sites in tubulin. Thus, they were able both, to experimentally validate part of their computational predictions and to identify new potential ligands. The whole approach, i.e., computational and crystallographic fragment screening, identified a total of 27 distinct binding sites on tubulin, including the major binding sites of known tubulin-drugs. This analysis revealed 11 novel sites (four on α-tubulin and seven on β-tubulin), that are not targeted by any of the structurally characterized tubulin-binding drugs or protein partners (Mühlethaler et al., 2021).

Unlike natural substances, which account for a large proportion of anti-tubulin drugs and are often chemically complex, fragments may represent favorable starting points for the rapid generation of synthetic anti-tubulin lead-like small molecules. Based on different criteria such as the novelty of the binding site, structural considerations and the importance of the molecular interactions between the fragment and the site, as well as the feasibility of synthesizing chemical derivatives, they produced different derivatives, so as to ‘grow’ the fragments with the objective of occupying the site cavity as well as possible. They then tested these derivatives for their ability to affect cell viability. While the fragments selected as starting points were not cytotoxic, some of the derivatives showed cell toxicity at micromolar concentrations. They further conducted a second round of chemical optimization and obtained an active fluorinated derivative, with a molecular weight of 377 Da, which they called Todalam. Todalam has a particular binding site located between two longitudinally aligned tubulin dimers, part of the Todalam binding site being located on α−tubulin, the other on β-Tubulin. Todalam has an inhibitory effect on *in vitro* tubulin assembly, disrupts microtubule networks in cells and arrests cells in G2/M. Thus, using a multidisciplinary approach combining structural biology, computational modelling, fragment screening, bio-guided and structure-guided chemical synthesis, the proof of concept that it is possible to synthesize a small molecule tubulin inhibitor that binds to a new, not yet described tubulin site has been achieved (Mühlethaler et al., 2022). Although this approach gave promising results, it remains to be explored whether all the identified sites represent targets for drug development.

### And then, united they stand

What if, instead of targeting a single tubulin site, it were interesting to target two different sites in order to increase therapeutic efficacy?

This is what our team has demonstrated. We were searching for agents capable of acting in synergy with paclitaxel (PTX) with the goal of reducing PTX doses and thereby its toxicity and the emergence of resistance. To that aim, we have screened an original collection of several thousand compounds using a cytotoxicity assay and selected a derivative of the carbazolone series (Carba1) able to sensitize cells to a low, non-toxic dose of PTX. While searching for the Carba1 mechanism of action, we were surprised to discover that this compound was able to bind tubulin at the colchicine site and, *in vitro*, to induce a dose-dependent decrease of the rate of tubulin polymerization (Peronne et al., 2020). How is it, then, that a MT depolymerizing agent synergizes with a MT stabilizing agent? The answer came from studies conducted in A. Akhmanova’s lab, in Utrecht. Using fluorescent PTX analogs, this team has identified the presence of taxane accumulation zones at MT ends that are transitioning from growth to depolymerization. They found that low non-saturating concentrations of a MT depolymerizing agent, which enhances catastrophes, promote taxane binding to growing MT tips (Rai et al., 2020). In collaboration with this team, we found that such a mechanism is at work with Carba1, thus explaining the observed synergy (Peronne et al., 2020).

Interestingly, the synergistic effect of Carba1 with PTX on tumor cell viability was also observed *in vivo* in xenografted mice (Peronne et al., 2020).

What happens at the structural level is not completely understood and is probably complex. It has been shown that taxanes bind to microtubules preferentially at their growing ends and that this binding is highly dependent on the conformational state of these ends. Notably, taxanes would preferentially bind to incomplete microtubule structures present at the ends. It thus can be assumed that the binding of a few molecules of a depolymerizing drug to the end of the MT induces conformational changes of this end, such as the formation of incomplete tubulin structures, e.g., tubulin sheets, promoting taxane-site PTX binding and MT stabilization (Rai et al., 2020). Nonetheless, we have demonstrated that this new mechanism favoring paclitaxel binding to dynamic MT can be transposed to *in vivo* mouse cancer treatments, paving the way for optimizing MTD-based cancer therapies, combining low doses of MT targeting agents with opposite mechanisms of action.

### In search of isotype-specific ligands

Several α- and β-tubulin genes exist producing different tubulin isotypes, which are very similar but non-identical (Little and Seehaus, 1988; Khodiyar et al., 2007; Kavallaris, 2010). These isotypes are expressed in different levels in different tissues, and at different stages in development. Moreover, altered tubulin isotype composition of microtubules is emerging as a feature of aggressive and teatment-refractory instead of treatment refractory cancers (Kavallaris et al., 1997; Kavallaris, 2010; Parker et al., 2017). For instance, it is well known that the upregulation of the β-III tubulin isotype is an important resistance mechanism that emerges in all chemotherapies employing MTDs (Dumontet and Jordan, 2010; Prassanawar and Panda, 2019). A possible molecular explanation of this mechanism has been provided by Susan Horwitz’s team. They conducted a comparative analysis of the binding of a radioactive paclitaxel analogue to different tubulin isotypes and identified a unique Ala218 in βIII-tubulin. Additional molecular dynamics simulations indicated that this residue could influence the ability of PTX to interact with its binding site (Yang et al., 2016a).

Ixabepilone, an analog of epothilone B, that preferentially suppresses βIII-tubulin dynamics has been reported to be more potent against cells expressing elevated βIII-tubulin and to be active in taxane-resistant metastatic breast cancer cells (Dumontet et al., 2009). The recent co-crystallization of this compound with tubulin has not yet explained its preferential action on βIII-tubulin (Xiao et al., 2021).

An attempt to selectively target the most divergent β tubulin isotype, known as β1 class VI (β VI-tubulin isotype), has been recently described. This isotype is found in megakaryocytes and platelets as well as in other blood cells. Using a fluorescence-based competition assay the authors compared the binding of different colchicine-site ligands to chicken brain tubulin and chicken erythrocyte tubulin, that contain almost exclusively chicken βVI tubulin. They found that many of the benzimidazole class of ligands have increased affinity for erythrocyte tubulin relative to brain tubulin and concluded that these findings may contribute to the development of drugs for cancers of various hematologic tissues (Montecinos et al., 2022).

In addition to the α/β tubulin heterodimer and the different isotypes, another member of the tubulin superfamily is γ-tubulin, which forms a ring-like template that initiates MT nucleation *in vivo*. Several studies have suggested that γ-tubulin might be a good candidate for the development of new anticancer drugs [for review see (Dráber and Dráberová, 2021)]. Because γ-tubulin is structurally quite similar to β-Tubulin, several teams have attempted to develop specific inhibitors of γ-tubulin from known drugs that bind to the colchicine binding site on β-Tubulin. This approach identified gatastatin as an inhibitor with higher affinity toward γ-tubulin compared with αβ-Tubulin (Chinen et al., 2015; Shintani et al., 2020). However, the cytotoxicity of gatastatin to cancer cells was relatively weak compared with that of conventional MTD, such as paclitaxel or vinblastine. Improved cytotoxicity was obtained when gatastatin was given in combination with polo-like kinase 1 (PLK1) inhibitors, suggesting that this combination may have therapeutic efficacy in cancer treatment (Ebisu et al., 2021).

## Towards evolutionary medicine: Targeting microtubules, but differentially

### When exploiting differences in tubulin structure allows targeting a parasite species while protecting the human host.

Although α- and β-tubulin are highly conserved proteins, the effects of microtubule-binding drugs vary in organisms belonging to distinct evolutionary groups. For example, plant tubulin and Apicomplexan tubulins have a much lower affinity for colchicine than animal tubulin (Morejohn and Fosket, 1991). In contrast, small synthetic molecules such as dinitroanilines bind specifically plant and Apicomplexa tubulins but not vertebrate or fungi ones (Morejohn et al., 1987; Hugdahl and Morejohn, 1993; Fennell et al., 2008; Lyons-Abbott et al., 2010). These observations imply that some structural differences do exist in the architecture of drug-binding sites on tubulin from plants and Apicomplexa on the one hand and mammalian tubulin on the other hand. These differences could be theoretically exploited to develop Apicomplexa (such as the parasites *P. falciparum* and *T. gondii*) -specific anti-tubulin drugs. The development of such drugs has been long hindered, however, by a lack of structural information on Apicomplexa and plant tubulins.

Focusing on conserved regions in plants, our team, in collaboration with modelling scientists, attempted to predict the structure of *P. falciparum* tubulin by homology modelling, using the crystal structures of bovine and porcine tubulins as models. We identified a “dockable” cleft corresponding to the oryzalin binding site **.** A virtual library of more than 300,000 chemical compounds was then screened for its ability to dock into the identified cleft. This approach allowed the selection of 3,023 compounds. We then picked 82 out of these 3,023 molecules and tested them directly on tobacco BY2 cells for their ability to depolymerize plant cell MTs, while having no detectable effect on mammalian MTs. We selected three compounds, that were also able to inhibit whole plant growth. These compounds did not show any significant effect on the assembly kinetics of bovine brain tubulin whereas they affected the assembly of commercially available soybean tubulin (Cytoskeleton Inc, Denver, CO, United States). In addition, these compounds exhibited a moderate cytotoxic activity towards *T. gondii* and *P. falciparum*, highlighting the potential of such novel herbicidal scaffolds in the design of future antiparasitic drugs (Soleilhac et al., 2018).

Recently, a more direct approach has been conducted by the Swiss team of the structural biologist M. Steinmetz and N. Morrissette (Irvine, California, United States), a specialist of apicomplexan parasites. Reasoning that the abundant tubulin of the *Tetrahymana thermophila* ciliate had a sequence very similar to that of the Apicomplexa, they used X-ray crystallography and cryo-electron microscopy to resolve the structure of the tubulin of *T. thermophila*. The comparison of the details of all six known drug-binding sites on mammalian tubulin with the equivalent regions of *T. thermophila* tubulin allowed them to observe that the most prominent differences between *T. thermophila* and human tubulin were located in the colchicine site. They thus tested three well-characterized colchicine site ligands, known to bind to distinct zones of the large colchicine site (colchicine, combretastatin A4, and plinabulin), for their ability to inhibit *T. thermophila* growth. Colchicine was found to have no effect on the growth of *T. thermophila*, whereas combretastatin A4 showed a partial growth inhibition activity and plinabulin had the strongest effect. This information and structural modeling guided them to identify candidate drugs that could discriminate between protozoan and vertebrate tubulins, from a commercially available ligand library. The molecules showing the most favorable binding poses were selected for *in vivo* testing. Among the various compounds tested, only one compound, which they named parabulin, inhibited *T. thermophila* growth. Finally, they showed that parabulin also inhibited the growth of the apicomplexan parasite *T. gondii* in human cells with an IC_50_ in the micromolar range, without harming the human host cells (Gaillard et al., 2021).

Thus, based on the structural differences between human and parasite tubulin, these two approaches have demonstrated the possibility of targeting only parasite tubulin, paving the way for the development of much-needed new parasite inhibitors.

## All roads lead to microtubules: Targeting microtubules, but unintentionally

Over the years, several enzymes have emerged as key factors implicated in human diseases. Cellular kinases, for instance, regulate various cell functions, and deregulation of some kinase activities have been involved in the etiology of numerous diseases, such as malignant, inflammatory or neurodegenerative disorders. Thus, kinases are the focus of intense drug discovery research, and the US Food and Drug Administration (FDA) has so far approved 68 small-molecule kinase inhibitors (Roskoski, 2022).

During the development of kinase inhibitors, *in vitro* profiling of adverse drug reactions is routinely performed. Panels of kinases are screened to get insight into the selectivity of the inhibitor and targeting several kinases is often indicative of potential toxicities leading to adverse reactions. However, the non-kinase targets that could contribute to the desired activity are rarely explored. Such non-kinase targets might cause or contribute to the cytotoxic/antiproliferative effects of anti-cancer kinase inhibitors. These non-kinase targets often remain undiscovered, as the antiproliferative effect of the kinase inhibitor is attributed to the inhibition of the targeted kinase (Munoz, 2017). The advent of automated microscopy methods, such as high content screening, allowed pharmaceutical companies to identify such off-targets during the process of drug development. Thus, the biopharmaceutical company Brystol Myers Squibb (BMS), while developing a series of LIM Kinase (LIMK) inhibitors based on an aminothiazole scaffold, found that some of these compounds (LIMKi 2 and LIMKi 5) depolymerized MTs, independently of their effect on LIMK, which was responsible of their cytotoxicity (Ross-Macdonald et al., 2008).

Such “off-target” effect has also been described after clinical trials have begun. Indeed, an increasing list of anticancer kinase inhibitor drugs, many of them in clinical trials, also turned out to bind tubulin at the colchicine site, acting as MT depolymerizing agents (reviewed in (Munoz, 2017; Tanabe, 2017; Steinmetz and Prota, 2018)). In 2013 for instance, Tivantinib, a non-competitive ATP inhibitor of the receptor tyrosine kinase MET, was also found to induce microtubule depolymerization in cells. This explains why Tivantinib was cytotoxic for cancer cells regardless of MET expression levels (Basilico et al., 2013; Katayama et al., 2013; Aoyama et al., 2014). Later, a structural analysis confirmed that this compound binds to tubulin at the colchicine site (Wang et al., 2016). Similarly, tubulin was found to be the prime target of several kinase inhibitors such as Rigosertib, originally identified in a screen for inhibitors of PLK1 (Jost et al., 2017) or Buparlisib, a highly selective inhibitor of the class I phosphoinositide 3-kinases (PI3K) (Bohnacker et al., 2017).

Why do so many kinase inhibitors also bind to tubulin? An explanation proposed by Steinmetz and Prota is that the colchicine site is a fairly deep, large binding pocket on tubulin, predominantly hydrophobic in nature, which could explain that several different types of anticancer drugs can also bind to tubulin (Steinmetz and Prota, 2018). There may indeed be some similarity between the binding site of drugs on kinases and on tubulin, as nocodazole, a tubulin depolymerizing agent that binds to the colchicine site, has also been shown to have high affinity for several kinases (Park et al., 2012).

It is remarkable that kinase inhibitors with such dual activity, i.e., also targeting microtubules, are all depolymerizing agents. Certainly, the most likely cause is that the colchicine site is large and can accommodate the binding of many molecules. Another possibility is that while the high content screening approaches mentioned above can easily detect depolymerization of cellular microtubules (Ross-Macdonald et al., 2008; Hoque et al., 2018), they are much less suitable for detecting a stabilized MT network on the basis of its morphology. Indeed, with the exception of stabilization by PTX which, at high doses, induces the formation of characteristic MT bundles, a stabilized MT network has a morphology similar to that of a normal network. To solve that problem, our team has developed a luminescent quantitative cell-based assay, suitable for automation, which allows the detection of stabilized microtubules without the need of microscopic examination (Ramirez-Rios et al., 2020). Using this assay to screen a kinase inhibitor library we found that several known kinase inhibitors have the ability to potently stabilize cellular microtubules. This includes for instance, selonsertib, an apoptosis signal-regulating kinase 1 (ASK1) inhibitor (Loomba et al., 2018) indicated for the treatment of non-alcoholic steatohepatitis (NASH), nintedanib and masitinib, which are both inhibitors of tyrosine kinase receptors (Dubreuil et al., 2009; Papadopoulos and Lennartsson, 2018). Masitinib is currently being investigated for the treatment of lateral amyotrophic sclerosis and various cancers (Trias et al., 2016; Scott, 2017; Waheed et al., 2018; Mora et al., 2019). Nintedanib is clinically indicated to treat idiopathic pulmonary fibrosis (Richeldi et al., 2014; Crestani et al., 2019). It is also currently approved for use in combination with docetaxel for treating advanced lung cancer that has progressed after first-line chemotherapy (Reck et al., 2014). Although we further demonstrated that none of the compounds interact directly with tubulin, their potent stabilizing effect on cellular MTs may account for their therapeutic effect as well as for some of their adverse side effects (Ramirez-Rios et al., 2020).

That MTs may be an off-target or even a primary target is true for other drugs than those targeting kinases. For example, it has recently been shown that the antipsychotic drug clozapine, the only effective drug for treatment-resistant schizophrenia, not only targets a wide range of monoamine receptors, but can also bind to tubulin and induce the depolymerization of cellular microtubules. This could contribute to the serious non-neurological effect, observed after treatment with clozapine, such as the development of agranulocytosis (Hino et al., 2022). Furthermore, this discovery reinforces the idea that microtubules represent an interesting therapeutic target for the treatment of schizophrenia (Marchisella et al., 2016).

It has now been demonstrated that bortezomib, a widely used 26S proteasomal subunit inhibitor, with anti-tumor activity in hematological malignancies, also has a stabilizing effect on MTs (Staff et al., 2013; Meregalli et al., 2014; Malacrida et al., 2021). This effect can account for bortezomib anti-cancer activity. Moreover, one major side effect of bortezomib is peripheral neuropathy, a painful axonal sensory–predominant and axon length-dependent peripheral neuropathy that affects ∼40% of bortezomib-treated patients (Meregalli et al., 2014; Kishimoto et al., 2019). The pathogenic mechanisms leading to such peripheral neuropathies were largely unknown until the recent work of F. Bartolini’s team at Columbia University (Pero et al., 2021). They showed that bortezomib caused axonopathy and disrupted mitochondria motility by increasing a post-translationally modified form of tubulin, delta2-tubulin, which is a marker of hyper-stable microtubules (Paturle-Lafanechere et al., 1994; Lafanechere and Job, 2000).

Hence, a whole range of drugs initially developed to target a specific mechanism, turn out to have a more general action through their effect on microtubules which may contribute to their therapeutic effect. Clearly, as with any drug, the effects on the organism depend on the dose, pharmacokinetics and pharmacodynamics of the drug administered. However, knowledge about the off-targets of these drugs is obviously useful for interpreting the results of clinical studies, for anticipating possible adverse effects and for the process of designing drug combinations.

Targeting different mechanisms simultaneously to increase the therapeutic efficacy of a drug can also be an intentionally implemented approach. For example, given the synergistic effect observed in combination therapies used in cancer treatment, involving tubulin-targeted drugs and other antitumor agents such as histone deacetylases inhibitors, DNA-damaging agents, or topoisomerase inhibitors, many efforts are now made to design of dual-target tubulin inhibitors [(Huang et al., 2019; Wu et al., 2020; Noha et al., 2021; Hauguel et al., 2022); and for a recent review see (Shuai et al., 2021)]. Compared to combination therapies such dual drugs may theoretically offer some advantages such as overcoming drug-resistance, superior treatment compliance, and lower risk of drug-drug interactions.

## Towards a surgical strike of diseased cells only

### Addressing the drug to the tumor

One way to address MTD directly to the tumor is to conjugate them to monoclonal antibodies targeting antigens expressed by cancer cells. The ultimate goal of such antibody–drug conjugates (ADCs) is to increase the therapeutic index of cytotoxic drugs, by the selective delivery of the chemotherapy to the tumor, whilst sparing the healthy tissues, thus avoiding undesired side effects. ADCs are a rapidly expanding class of anticancer therapeutics. The ideal properties sought for ADCs, the obstacles encountered, the solutions found are outside the scope of this review and have been the subject of several reviews to which the reader may be referred (Donaghy, 2016; Chen et al., 2017; Abbas et al., 2021; Dean et al., 2021).

Of the ADCs approved by the FDA for clinical use, the Ado-trastuzumab emtansine (Kadcyla^®^), also called T-DM1, is a conjugation of a tubulin depolymerizing agent, derivative of maytansine, to the anti-HER2 antibody trastuzumab. T-DM1 has been approved for the treatment of HER-2 positive metastatic breast cancers (Nicoletti et al., 2015). SGN-35 is also an approved ADC, which consists of the drug MMAE, a dolastatin-derivative targeting the vinca alkaloid site, coupled to an antibody recognizing the tumor marker CD30 (Chen et al., 2017).

However, these ADCs are still far from the concept of an ideal targeted therapy and resistances to these ADCs remains a challenge, as well as undesirable specific toxicities (ocular, hepatic). These ADCs also often suffer from issues associated with intratumor heterogeneity.

Thus, several other ADCs, potentially more potent and selective are currently under development or in clinical trials (Chen et al., 2017; Dean et al., 2021). These approaches include the development of ADCs with dual payloads that could tackle tumor heterogeneity and the occurrence of resistances (Yamazaki et al., 2021).

### Colchicine and inflammatory diseases: When hitting liver cells alone generates a systematic therapeutic effect.

An unexpected local pharmacology has been recently described that explains the well-known anti-inflammatory effect of colchicine. Colchicine was one of the very first drugs identified as targeting tubulin. In fact, it was rather the opposite: it was because tubulin behaved as a colchicine-binding protein that it was isolated and identified as the constituent protein of microtubules by G. Borisy and E. Taylor in 1967 (Borisy and Taylor, 1967). Extracts of Colchicum autumnale have long been used in traditional medicine. Pure colchicine is still used today for the treatment of inflammatory diseases such as gout, pericarditis or familial Mediterranean fever. More recently, it has been shown that a low-dose of colchicine combines anti-inflammatory action with a favorable safety profile in COVID-19 patients (Deftereos et al., 2020; Schlesinger et al., 2020). The narrow therapeutic window of colchicine limits its use for the treatment of other diseases such as cancers (Finkelstein et al., 2010).

It has long been thought that colchicine acts by depolymerizing the microtubules of circulating myeloid cells, preventing their recruitment to the tissues where inflammation occurs. However, this interpretation could not be reconciled with the fact that only the microtubules of myeloid cells are targeted; indeed, colchicine at safe effective doses lacks anti-mitotic side effects, such as neutropenia and alopecia. Moreover the concentration of colchicine measured in the plasma of patients after administration of an effective dose is much lower than the concentration necessary *in vitro* to block neutrophil chemotaxis (Weng et al., 2021).

An elegant explanation for this paradox was recently provided by T. Mitchison’s team at Harvard Medical School. They demonstrated, in mice, that colchicine accumulates selectively in the liver where it depolymerizes the microtubules of hepatocytes only. Indeed, hepatocytes are specialized in the clearance of xenobiotic compounds and express drug transporters that concentrate them. This selective accumulation of colchicine in hepatocytes induces Nrf2 activation, leading to secretion of hepatokines, which, via the bloodstream, will exert a systemic anti-inflammatory action (Weng et al., 2021). Thus, the locally occurring accumulation of colchicine in the liver, is central to the colchicine therapeutic action. Although this new, indirect mode of action of colchicine has not yet been validated in humans, the concept that a molecule can systemically modulate the immune system by inducing hepatokines has broad implications for the pharmacology of compounds which enter the blood and act in the liver (Shi et al., 2021).

### Perspective: Exploiting the differences between diseased and normal cells/organs

In cells, MT dynamics is tightly regulated by a balance between the activities of MT stabilizing and destabilizing proteins (Lieuvin et al., 1994; Andersen, 2000; Gache et al., 2005; Komarova et al., 2005; Niethammer et al., 2007). For instance, the interaction of the microtubule growing tip with proteins such as CLIP-170 results in microtubule stabilization (Small and Kaverina, 2003; Akhmanova and Hoogenraad, 2005). The binding of such proteins to tubulin is tightly regulated either by their phosphorylation/dephosphorylation (Cassimeris and Spittle, 2001; Johnson and Stoothoff, 2004) or by tubulin modifications such as the modifications induced by the tubulin tyrosination cycle (Peris et al., 2006, 2009; Verhey and Gaertig, 2007; Barisic and Maiato, 2016; Pero et al., 2021). Targeting a regulatory protein of MT dynamics which is more expressed or more active in diseased cells or in tumors represents an interesting alternative therapeutic strategy. Indeed, this could improve the selectivity of the drug towards diseased organs or tumors and thus offer a significant therapeutic window.

LIM Kinases (LIMKs) are such regulatory enzymes. They regulate the architecture of the actin cytoskeleton by phosphorylation and inactivation of cofilin (Scott and Olson, 2007). LIMKs also regulate microtubule dynamics (Gorovoy et al., 2005; Prudent et al., 2012; Ray et al., 2014; Mardilovich et al., 2015), but whether this regulation occurs through a direct binding of LIMKs to microtubules (Bhardwaj et al., 2014) or through phosphorylation of an associated protein is still under debate (Pleines et al., 2013; Prunier et al., 2017). When LIMKs are inhibited, microtubules are stabilized and actin microfilaments are severed and disorganized (Prudent et al., 2012; Mardilovich et al., 2015). Interestingly, expression of LIMKs or cofilin phosphorylation are elevated in numerous tumors (Davila et al., 2003; Okamoto et al., 2005; Bagheri-Yarmand et al., 2006; Ahmed et al., 2008; McConnell et al., 2011; Manetti, 2012; Park et al., 2014). In breast cancer models, activation of LIMKs is the last step of an integrin-linked machinery of cytoskeletal regulation that enables tumor initiation and metastatic colonization (Shibue et al., 2013). Thus, LIMKs are enzymes whose activity is elevated in cancers compared to normal tissue. Consequently, their inhibition could selectively target tumors. Indeed, inhibition of LIMK activity using RNAi or pharmacological inhibitors efficiently reduced the growth of tumor cells and their pro-invasive properties *in vitro* (Li et al., 2013a; Prunier et al., 2016a). Also, tumors expressing dominant-negative LIMK1 grow more slowly and are less metastatic in mice (Li et al., 2013a). However, contradictory results have been obtained in animal models with different pharmacological inhibitors of LIMKs. Thus, the Pyr1 inhibitor shows strong anti-tumor activity in a leukemia model (Prudent et al., 2012) and in various breast cancer models (Prunier et al., 2016a). In contrast, a LIMK inhibitor developed by BMS (BMS3 or LIMKi3), has no anti-tumor effect when administered *in vivo* to mice bearing xenografted breast cancer tumors (Li et al., 2013b). These differences may be explained by differences in the selectivity profile of the inhibitors, by differences in the pharmacokinetics and pharmacodynamics of the inhibitors, or by differences of the sensitivity of the cell lines xenografted into the mice.

Interestingly, the Pyr1 inhibitor did not induce detectable adverse effects in these animal models, contrary to PTX, and was found active also on PTX -resistant tumors (Prunier et al., 2016b; 2016a), indicating that such a therapeutic strategy could represent a useful alternative for cancer treatment.

Cytoskeleton-related neuronal defects are landmarks of psychiatric disorders, in particular schizophrenia (Benitez-King et al., 2004; Fromer et al., 2014; Marchisella et al., 2016). Moreover, in patients with schizophrenia, LIMK was found to be deregulated (Datta et al., 2015; Zhao et al., 2015), making this enzyme a potential pharmacological target. This hypothesis has been tested by administration of the Pyr1 inhibitor in MAP6 KO mice, an animal model useful for the study of psychiatric disorders, particularly schizophrenia (Andrieux et al., 2002; Begou et al., 2008; Fournet et al., 2012; Volle et al., 2012; Jonckheere et al., 2018). It was found that chronic LIMK inhibition by long-term treatment with Pyr1 can restore synaptic plasticity and alleviates some behavioral defects in MAP6 KO mice, thus validating the hypothesis that modulation of LIMK activity could represent a new therapeutic strategy for neuropsychiatric diseases (Gory-Fauré et al., 2021).

The detyrosination-tyrosination cycle of tubulin is another regulatory mechanism of MTs (Barra et al., 1988; MacRae, 1997; Janke and Bulinski, 2011; Nieuwenhuis and Brummelkamp, 2019). This cycle begins with the cleavage of the C-terminal tyrosine of α-tubulin by a carboxypeptidase (TCP) and the re-addition of this amino acid to the carboxy-terminus by a ligase, the tubulin tyrosine ligase (TTL). The tyrosination reaction is specific for tubulin, it has never been observed for any other known protein. The enzymatic cycle generated by the successive action of TCP and TTL generates two forms of tubulin, tyrosinated tubulin and detyrosinated tubulin. A third form of tubulin, delta2-tubulin, lacking the two last C-terminal amino acids, is an irreversible tubulin post-translational modification and a marker of hyperstable MTs (Paturle-Lafanechere et al., 1991, 1994; Lafanechere and Job, 2000).

Tyrosinated tubulin and detyrosinated tubulin are important regulatory signals for mitosis (Badin-Larçon et al., 2004; Peris et al., 2006; Barisic et al., 2015), neuronal physiology (Konishi and Setou, 2009; Marcos et al., 2009; Gobrecht et al., 2016) and muscle mechanotransduction (Belmadani et al., 2004; Kerr et al., 2015; Robison et al., 2016). Consequently, abnormal levels of detyrosinated tubulin are associated with cell transformation and tumor aggressiveness (Lafanechere et al., 1998; Mialhe et al., 2001; Kato et al., 2004; Soucek et al., 2006; Seve et al., 2008; Whipple et al., 2010), neuronal disorganization, synaptic function and neuronal disease (Erck et al., 2005; Peris et al., 2022), heart failure and cardiomyopathies (Belmadani et al., 2004; Robison et al., 2016). Thus, pharmacological inhibition of TCP, to decrease the amount of detyrosinated tubulin and restore normal levels of tyrosinated tubulin should interfere with pathological processes (Chen et al., 2018). Previously, parthenolide, a poorly selective (Hotta et al., 2021) TCP inhibitor was discovered by screening a collection of a chemical library of natural extracts using a cell-based assay, which detects the abundance of detyrosinated tubulin (Fonrose et al., 2007). However, the search for new, more specific TCP inhibitors was hampered by a lack of knowledge about the identity of this enzyme. After more than 40 years of research, the molecular nature of this enzyme was recently discovered (Aillaud et al., 2017; Nieuwenhuis et al., 2017) and knowledge of its structure (Adamopoulos et al., 2019; Li et al., 2019; Wang et al., 2019) should now facilitate the search for more selective inhibitors. Yet, since then, another enzyme with TCP activity (Landskron et al., 2022) has been described, which broadens the field of research for such inhibitors and will require clarification of their therapeutic applications.

Besides the above-described examples, the list of new therapeutic targets is growing steadily - as for instance disrupting the microtubule-dependent machinery underlying cancer cell migration/adhesion to combat metastasis (Rafiq et al., 2019)—with the detailed knowledge of microtubule functions and their dysregulation in pathologies. This should make it possible, in the medium term, to offer doctors new, more targeted treatments.

## Discussion and conclusion

In this review, I have illustrated, on the basis of examples, the variety of pharmacological approaches currently in development to target microtubules.

The direct targeting of microtubules, with new chemical entities and at new sites, remains of major therapeutic interest, especially as an alternative to the emergence of resistance in cancer treatment. Moreover, the fact that different sites can interact may allow us to imagine original therapeutic combinations, which remain to be explored. Finally, having access to molecules with different, possibly weaker, affinities for tubulin may represent a therapeutic interest, both to limit the side effects and to be used in combitherapies. Such combination therapies may also be of interest for targeting tumor heterogeneity.

The discovery that some kinase inhibitors also target microtubule dynamics may help to predict/understand some of their adverse effects. These inhibitors may also represent novel scaffolds for the synthesis of agents that more selectively target tubulin. Moreover the develoment of dual-drugs may lead to improved therapeutic efficacy.

However, the growing knowledge of the different mechanisms by which microtubule functions are regulated and how they are altered in disease now allows for more targeted therapeutic strategies. In contrast to drugs that directly target tubulin, these promising approaches should reduce the adverse effects resulting from targeting other microtubule functions. The pioneering approaches of this type that we have carried out show increased therapeutic efficacy in animal models and no detectable adverse effects (Prudent et al., 2012; Prunier et al., 2016a; Gory-Fauré et al., 2021). Given the limitations of animal models, and the many pitfalls that drug development must overcome, one must necessarily be cautious. In fine, only a therapeutic improvement observed in the human clinic will validate these approaches.

Finally, this review represents only a partial snapshot of the current knowledge in this field. It is a landscape that is constantly reshaping itself, offering new perspectives. For instance, understanding not only how microtubules are deregulated in diseased cells, but also how they respond to changes in the diseased microenvironment should pave the way for exciting new therapeutic avenues.
